# Imaging 4D morphology and dynamics of mitral annulus in humans using cardiac cine MR feature tracking

**DOI:** 10.1038/s41598-017-18354-2

**Published:** 2018-01-08

**Authors:** Shuang Leng, Shuo Zhang, Meng Jiang, Xiaodan Zhao, Rui Wu, John Allen, Ben He, Ru San Tan, Liang Zhong

**Affiliations:** 1 National Heart Research Institute Singapore, National Heart Centre, Singapore, 5 Hospital Drive, 169609 Singapore; 2 Philips Healthcare, 622 Lorong 1, Toa Payoh, 319763 Singapore, Singapore; 3 Department of Cardiology, Renji Hospital, School of Medicine, Shanghai Jiaotong University, Shanghai, 200001 People’s Republic of China; 4 Duke-NUS Medical School, 8 College Road, Singapore, 169857 Singapore

## Abstract

Feature tracking in cine cardiac magnetic resonance (CMR) is a quantitative technique to assess heart structure and function. We investigated 4-dimensional (4D) dynamics and morphology of the mitral annulus (MA) using a novel tracking system based on radially rotational long-axis cine CMR series. A total of 30 normal controls and patients with mitral regurgitation were enrolled. The spatiotemporal changes of the MA were characterized by an in-house developed program. Dynamic and morphological parameters extracted from all 18 radial slices were used as references and were compared with those from subsequently generated sub-datasets with different degrees of sparsity. An excellent agreement was found among all datasets including routine 2-, 3- and 4-chamber views for MA dynamics such as peak systolic velocity (Sm) and mitral annular plane systolic excursion (MAPSE). MA morphology for size and shape was addressed adequately by as few as 6 radial slices, but poorly by only three routine views. Patients with regurgitation showed significantly reduced mitral dynamics and mild annular deformation, which was consistent between three routine views and 18 reference slices. In conclusion, feature tracking cine CMR provided a comprehensive and distinctive profile for 4D MA dynamics and morphology, which may help in studying different cardiac diseases.

## Introduction

Atrioventricular valves separate the atria and ventricles. The mitral annulus (MA) forms the perimeter of the left-sided atrioventricular valves. Its dynamic characteristics mirror left ventricular (LV) contraction, relaxation and filling, providing a window into structural and functional information for assessing LV systolic and diastolic function^1^. Hence, reliable quantification of MA deformation throughout the cardiac cycle is highly important. Tissue Doppler imaging (TDI), an echocardiographic technique based on the Doppler principle, has been widely adopted to measure the velocity of myocardial motion in clinical practice. A number of TDI-derived parameters, including peak velocity during systole (Sm), early diastole (Em), and late diastole (Am) have been shown to be potentially important prognosticators for various cardiovascular diseases^2^. In common with other Doppler modes, however, TDI is limited by angle dependency^3^ and influenced by translational motion and tethering^4^. Cardiac magnetic resonance (CMR) offers superior soft-tissue contrast and spatial resolution compared to echocardiography, potentially allowing improved quantification of MA deformation^5^. Most of the prior CMR studies were based on three routinely acquired long-axis cine imaging slices for MA motion tracking, with only six points for reconstruction of the 3-dimensional (3D) MA geometry^1,6^. Whether this is sufficient to constitute a good representation has hitherto not been studied. The obligatory minimum number of points or slices for satisfactory depiction of MA dynamics and morphology is not known.

The primary aims of this paper were: (1) to quantify the 4-dimensional (4D) dynamic and morphological characteristics of the MA using feature tracking in a series of equally spaced radially rotational long-axis cine CMR, based on which a reference set of clinically relevant parameters including peak systolic and diastolic velocities, displacement, 3D area, perimeter, diameter and height can be derived; (2) to systematically investigate the impact of different numbers of imaging slices on quantification accuracy by comparing results from sub-datasets with less equally spaced radial slices to the reference dataset; and (3) to study the difference in MA dynamics and morphology between normal controls and patients with mitral regurgitation.

## Results

### Basic Demographic Data

A total of 30 human subjects including 16 normal controls (mean age 54 ± 13 years) free from any objective evidence of cardiovascular or other diseases and 14 patients with mitral regurgitation (50 ± 18 years) were consecutively enrolled in this prospective study. The demographics and clinical characteristics of these two age- and gender-matched groups are given in Table 1. The patient group had significantly higher LV mass index compared to normal controls.

**Table 1:** Baseline demographic and clinical characteristics of study subjects.

Parameters	Controls (*n* = 16)	Patients (*n* = 14)	*P* Value
Male/Female	8/8	6/8	^—^
Age (years)	55.5 (48.3, 63.3)*	53.0 (42.0, 59.0)*	0.467
BSA (m^2^)	1.62 ± 0.13	1.81 ± 0.23	0.010
Height (cm)	162.5 ± 6.7	165.9 ± 7.7	0.156
Weight (kg)	57.6 ± 7.7	71.8 ± 16.2	0.006
LV EDV index (ml/m^2^)	70.7 ± 12.3	89.9 ± 49.2	0.631
LV ESV index (ml/m^2^)	28.0 ± 6.6	45.0 ± 44.4	0.631
LV SV index (ml/m^2^)	42.7 ± 7.5	42.9 ± 12.8	0.760
LV EF (%)	60.7 ± 5.2	58.8 ± 23.1	0.315
LV Mass index (g/m^2^)	43.2 ± 9.1	105.3 ± 35.0	<0.0001^#^
**Severity of mitral regurgitation (n)**			
- Mild	0	10	—
- Mild to moderate	0	1	—
- Moderate	0	1	—
- Moderate to severe	0	2	—

Data are represented as mean ± SD. BSA: body surface area; LV: left ventricular; EDV: end-diastolic volume; ESV: end-systolic volume; SV: stroke volume; EF: ejection fraction; *median (first quartile, third quartile). *P* values from Mann-Whitney U test. ^#^LV mass was significantly higher in the patient group than normal controls.

### Feature Tracking Cine CMR

**Figure 1:**
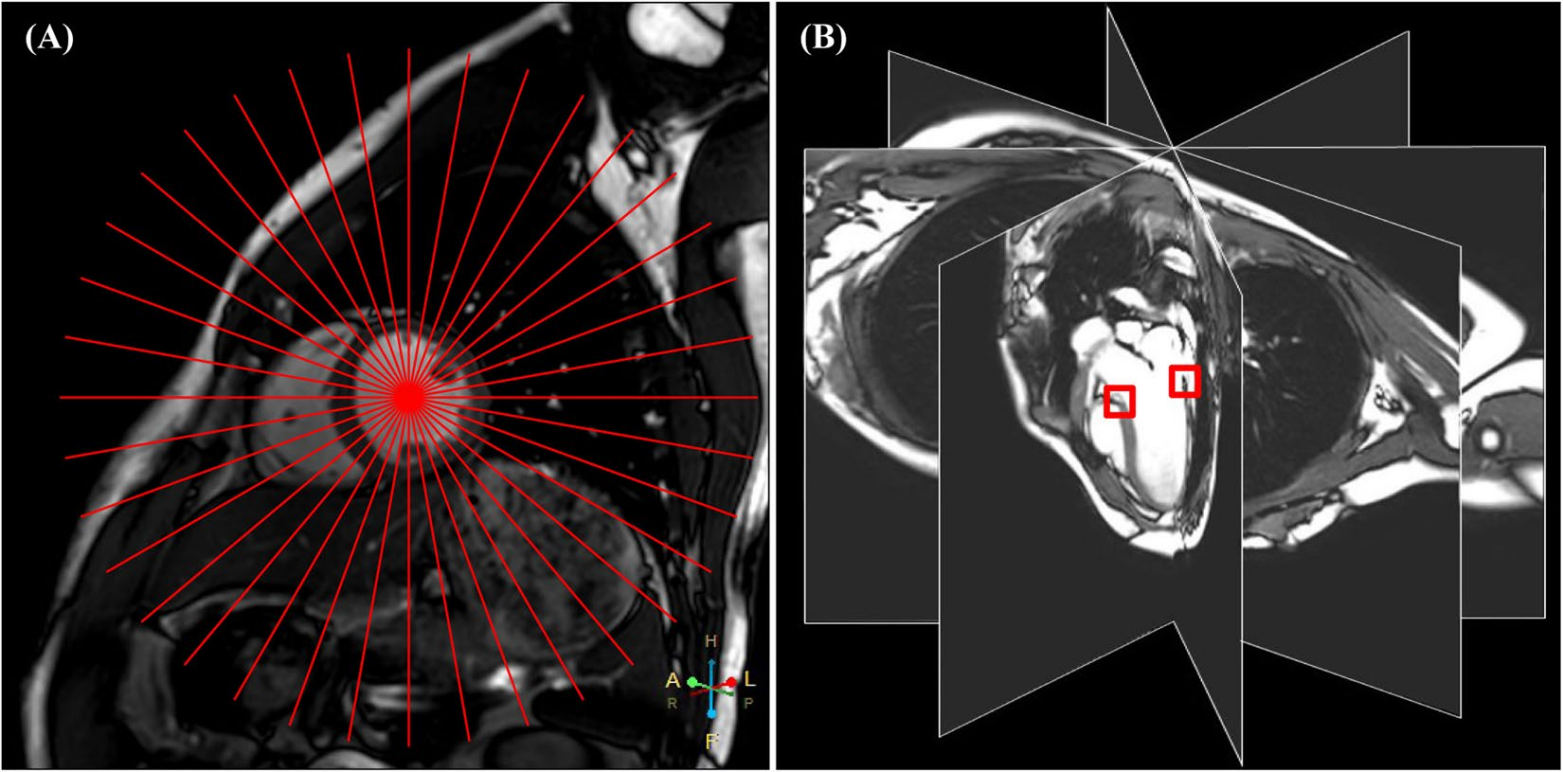
Radially rotational long-axis cine CMR. Localization of the 18 imaging slices in a short-axis view (red lines represent cutting planes) (**A**) and selected radial slices in 3D reconstructed view (**B**). Two mitral annulus points in each of the 18 acquired radial slices were tracked (red squares in (**B**)).

A total of 18 long-axis slices were obtained from cine CMR covering the mitral valve orifice with 10° angular equidistance in a radially rotational manner (Fig. 1A). Feature tracking was successfully performed in all 30 human subjects. From each acquired slice, the motion trajectories of two annular points were determined (Fig. 1B) over the entire cardiac cycle using the in-house developed semi-automatic algorithm for further parameter extraction (two typical examples can be seen in Supplementary Video S1 for a normal control and Video S2 for a patient with moderate to severe mitral regurgitation). The semi-automatic tracking process requires <1 min per MA point, including manual placement of the mask, tracking, manual correction if necessary, and visual review of tracking outputs.

In addition to the results obtained based on all 18 radial slices as reference, the same quantitative parameters were also generated from multiple sub-datasets with different degrees of sparsity consisting of less equally spaced radial slices. These included 9 slices (every other slice), 6 slices (every third slice), and 3 slices (routine 2-, 3-, 4-chamber views) for all subjects, to investigate the influence from the numbers of imaging slices. The average angular separations were about 67°, 52°, and 61° between 2- and 3-chamber views, between 3- and 4-chamber views, and between 4- and 2-chamber views, respectively, to best approximate the clinically conventional views.

### 4D Dynamics Based on Motion Estimation

**Figure 2:**
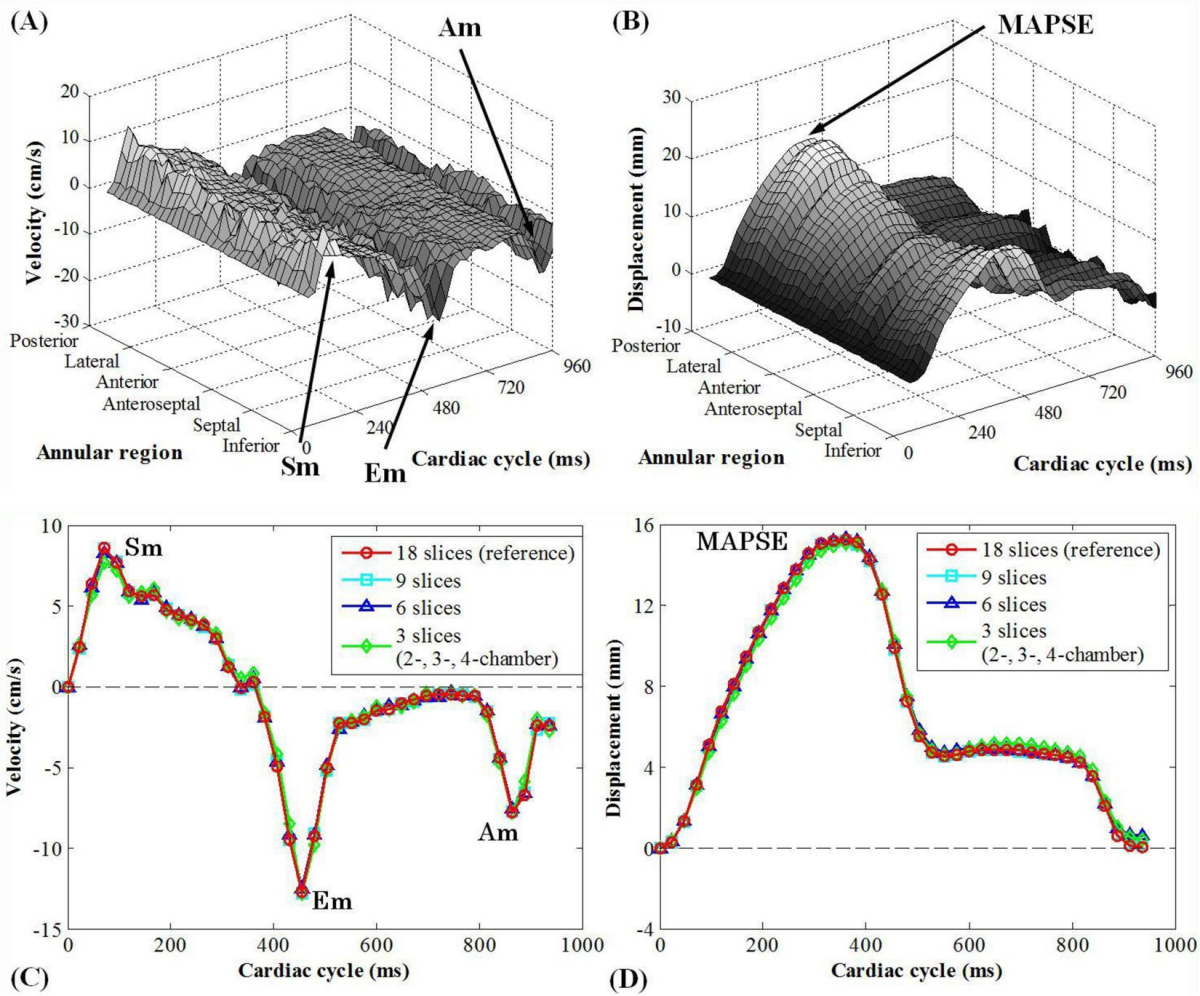
Mitral annulus dynamics obtained by feature tracking. In (**A**,**B**), the MA velocity and displacement were plotted as a function of annular region and cardiac cycle for all radially rotational slices and cardiac phases. In (**C**,**D**), the spatially averaged velocity and displacement curves were plotted along the cardiac cycle for 18, 9, 6, and 3 slices, respectively. For the 3-slice version, the clinical routine 2-, 3- and 4-chamber views were chosen. Data were obtained from a 39-year-old male normal control. Sm: peak systolic velocity; Am: peak late diastolic velocity; Em: peak early diastolic velocity; MAPSE: mitral annular plane systolic excursion.

By tracking the MA motion trajectory in the spatiotemporal dimension along the annular region for all cardiac phases, four dynamic parameters were obtained. These included peak systolic velocity (Sm), peak early diastolic velocity (Em), peak late diastolic velocity during atrial contraction (Am) and mitral annular plane systolic excursion (MAPSE). They corresponded to the maximal peaks in the velocity curves throughout the cardiac cycle and the maximal values in the displacement curves, respectively (Fig. 2A,B).

**Figure 3:**
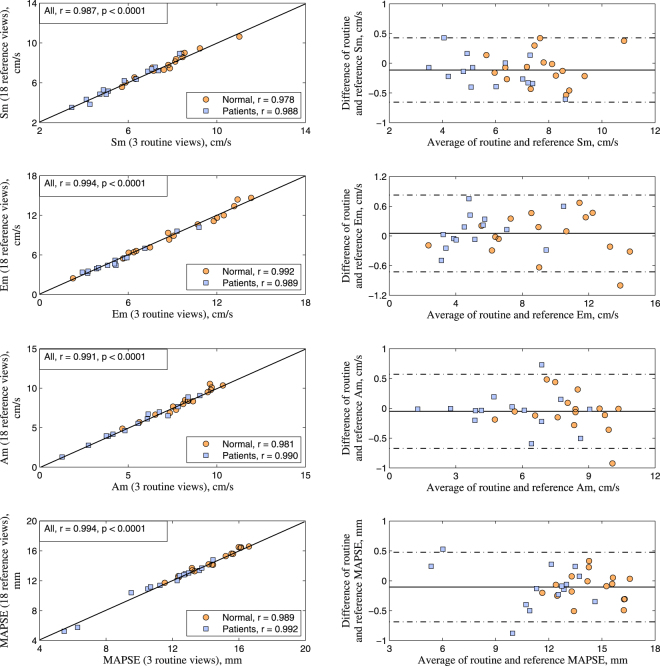
Comparison of mitral annulus dynamics based on long-axis cine feature tracking. Correlation and difference between results from 18 radially rotational slices and 3 clinical routine long-axis views are shown in scatter plot (left) and Bland Altman plots of difference against average (right) for all dynamics parameters Sm, Em, Am, and MAPSE. Normal controls indicated by circles and patients by squares.

Temporal MA dynamics agreed very well throughout the cardiac cycle for all sub-datasets, including the local maxima for Sm, Em, Am, and MAPSE (Fig. 2C,D). In particular, results from the three clinical routine long-axis views displayed excellent agreement with the reference values, reflected by the high correlations (*r* = 0.987, 0.994, 0.991, 0.994 for Sm, Em, Am and MAPSE, respectively, all *p* < 0.0001, Fig. 3 left), small bias and tight Bland Altman 95% limits of agreement (Fig. 3 right), as well as high intra-class correlation coefficient (ICC) values (0.993, 0.997, 0.995 and 0.997 for Sm, Em, Am and MAPSE, respectively). A complete comparison can be found in Supplementary Table S1.

### 4D Morphology Based on Geometry Reconstruction

**Figure 4:**
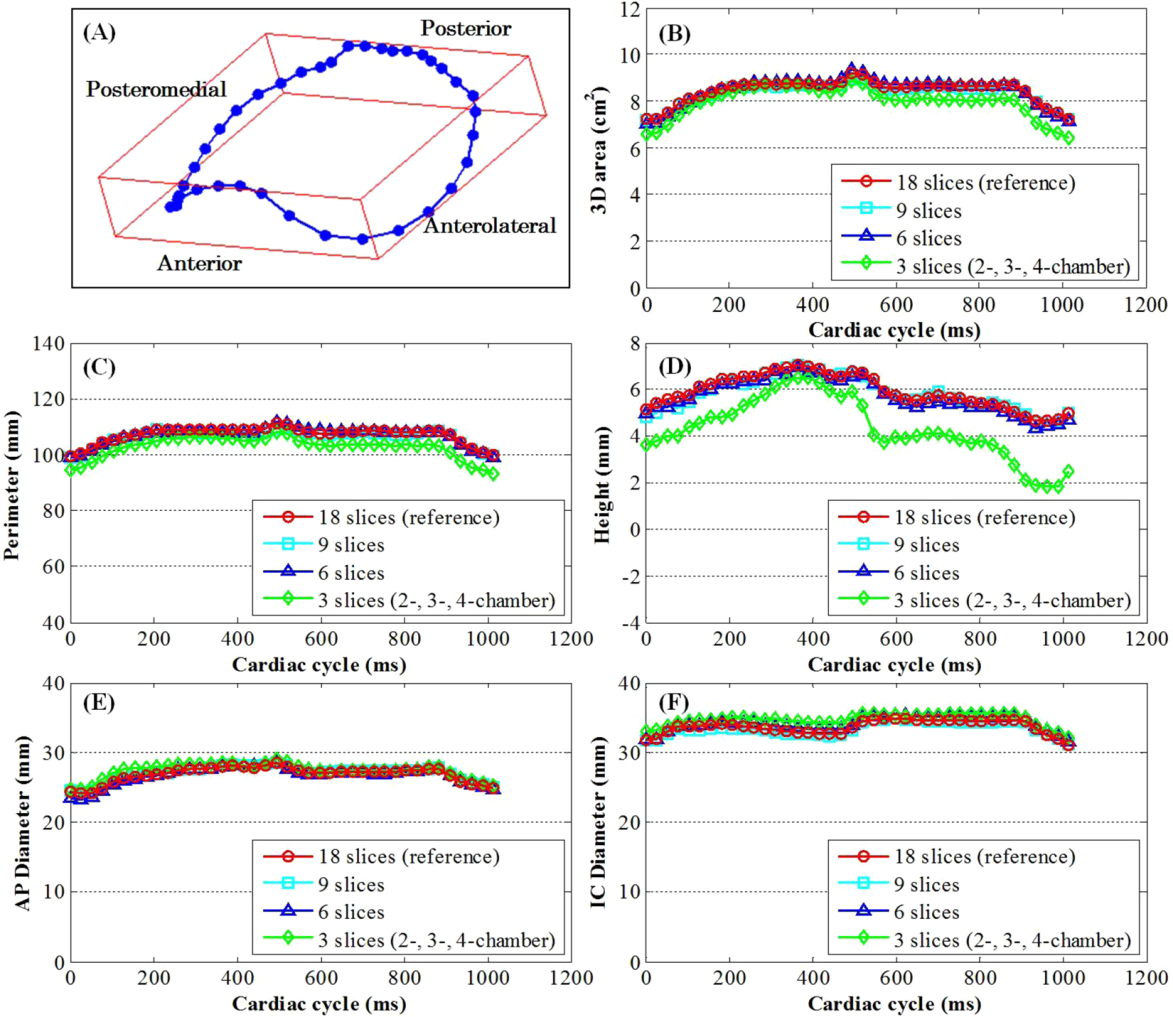
Mitral annulus morphology obtained by feature tracking and geometry reconstruction. 3D reconstructed annulus (**A**), and temporal changes of the quantitative parameters 3D area (**B**), perimeter (**C**), height (**D**), AP (**E**) and IC diameter (**F**) along the cardiac cycle for 18, 9, 6, and 3 slices, respectively. For 3-slice version, the clinical routine 2-, 3- and 4-chamber views were chosen. Data were obtained from a 49-yr-old female normal control.

**Figure 5:**
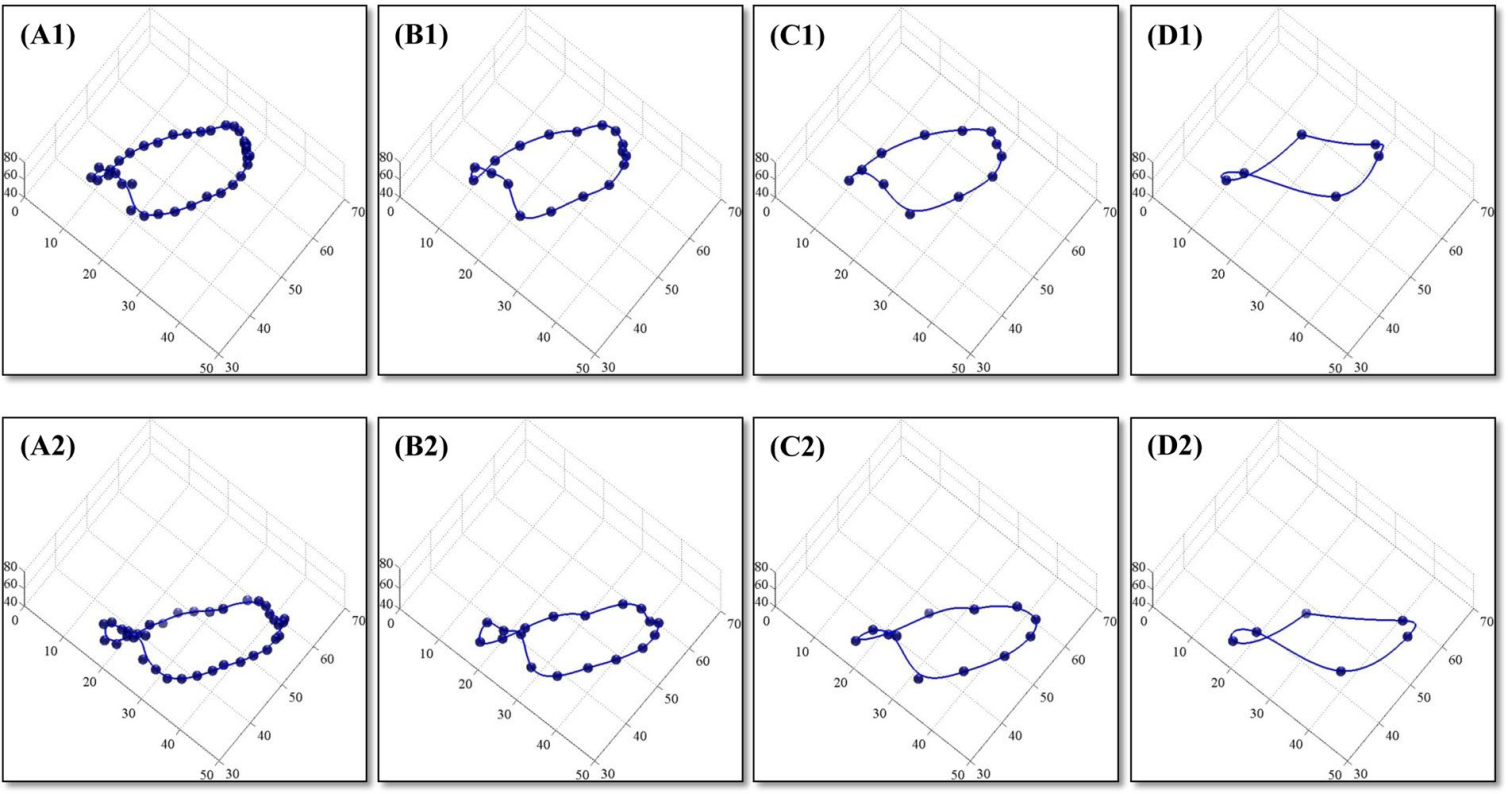
Mitral annulus morphology obtained by feature tracking and geometry reconstruction with different numbers of radially rotational long-axis slices. Reconstructed geometries of the mitral annulus are shown for (**A**) 18, (**B**) 9, (**C**) 6, and (**D**) 3 imaging slices at both end-diastole (top) and end-systole (bottom). Data were obtained from a 49-yr-old female normal control.

The geometry of a nonplanar, saddle-shaped MA was reconstructed based on feature tracking and spatial transformation of the imaging coordinates. A typical example is shown in Fig. 4A with all 18 radial slices for a chosen cardiac phase. Multiple morphological parameters were obtained, which included 3D area, perimeter, height, antero-posterior (AP) diameter, inter-commissural (IC) diameter and the ratio of AP to IC diameter. For comparison between different sub-datasets, Fig. 5 presents the qualitative geometric change of the reconstructed annulus at both end diastole (ED) and end systole (ES), while the quantitative change of the correspondingly derived parameters were plotted in Fig. 4B–F as a function of cardiac time. A full demonstration of the 4D MA morphology for different sub-datasets can be seen in Supplementary Video S3 for a normal control.

**Figure 6:**
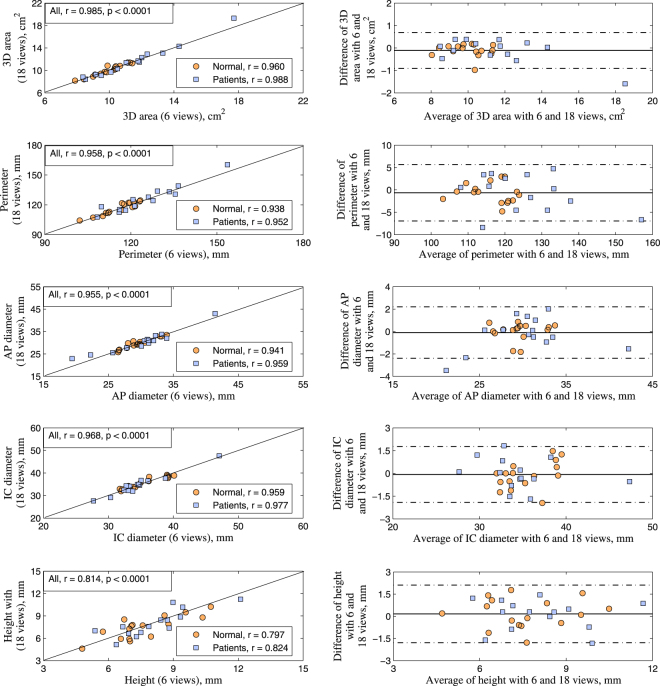
Comparison of mitral annulus morphology based on long-axis cine feature tracking and geometry reconstruction. Correlation and difference between results from 18 and 6 radially rotational long-axis slices are shown in scatter plot (left) and Bland Altman plots of difference against average (right) for all morphological parameters 3D area, perimeter, diameter, and height, respectively. Normal controls indicated by circles and patients by squares.

Significant agreement was found among MA morphological measurements from 18, 9 and 6 radial slices, as shown in the 45° line scatter and Bland Altman plots (Fig. 6) with excellent correlations (*r* = 0.985, 0.958, 0.955, 0.968, 0.814 for 3D area, perimeter, AP diameter, IC diameter and height, respectively, all *p* < 0.0001) and small limits of agreement. However, unlike dynamics, the MA morphology derived from three clinical routine long-axis views exhibited poorer correlation with larger deviation compared to those from the 18 reference slices. A complete comparison can be found in Supplementary Table S2.

### Comparisons between Controls and Patients

A comprehensive comparison of MA dynamics and morphology between normal controls and patients is presented in Tables 2 and 3. Based on the results from 18 reference slices, all dynamic parameters reduced significantly in patients, namely, Sm (5.9 ± 1.6 vs. 7.8 ± 1.3 cm/s, *p* = 0.003), Em (5.3 ± 2.2 vs. 9.3 ± 3.5 cm/s, *p* = 0.002), Am (5.6 ± 2.3 vs. 8.2 ± 1.6 cm/s, *p* = 0.002), and MAPSE (11.4 ± 2.8 vs. 14.5 ± 1.6 mm, *p* = 0.001). Data from three clinical routine slices, i.e. 2-, 3-, and 4-chamber views, showed the same results, in agreement to the reference. Meanwhile, 3 out of 6 morphological parameters increased significantly in patients, which included 3D area at ED (10.7 ± 3.2 vs. 8.7 ± 1.1 cm^2^, *p* = 0.014), perimeter at ED (120.7 ± 15.3 vs. 109.5 ± 7.4 mm, *p* = 0.011) and with temporal averaging (125.8 ± 13.5 vs 116.6 ± 6.5 mm, *p* = 0.031), as well as the ratio of AP to IC diameter at ED (0.85 ± 0.15 vs. 0.73 ± 0.10, *p* = 0.020). The patient group had also increased MA perimeter at ES (129.6 ± 12.3 vs. 120.4 ± 6.7 mm, *p* = 0.051) and AP diameter at ED (28.6 ± 5.9 vs. 25.0 ± 2.5 mm, *p* = 0.056) although the difference did not reach statistical significance. Similar results were also observed with 6 radial slices.

**Table 2:** Mitral annulus dynamics in patients with mitral regurgitation in comparison to those in normal controls.

Parameters	Controls (*n* = 16)		Patients (*n* = 14)		*P* Value	
	18 slices	3 slices*	18 slices	3 slices*	18 slices	3 slices*
Sm, cm/s	7.8 ± 1.3	7.7 ± 1.3	5.9 ± 1.6	5.8 ± 1.5	**0.003**	**0.002**
Em, cm/s	9.3 ± 3.5	9.3 ± 3.4	5.3 ± 2.2	5.4 ± 2.3	**0.002**	**0.002**
Am, cm/s	8.2 ± 1.6	8.1 ± 1.6	5.6 ± 2.3	5.5 ± 2.2	**0.002**	**0.002**
MAPSE, mm	14.5 ± 1.6	14.4 ± 1.6	11.4 ± 2.8	11.3 ± 2.7	**0.001**	**0.001**

Data were presented as mean ± SD. *Dynamic result based on 3 slices (clinical routine 2-, 3-, and 4-chamber views); *P* values obtained using Mann-Whitney U test. Sm: peak systolic velocity; Em: peak early diastolic velocity; Am: peak late diastolic velocity; MAPSE: mitral annular plane systolic excursion.

**Table 3:** Mitral annulus morphology in patients with mitral regurgitation in comparison to those in normal controls.

Parameters		Controls (*n* = 16)		Patients (*n* = 14)		*P* Value	
		18 slices	6 slices*	18 slices	6 slices*	18 slices	6 slices*
3D Area, cm^2^	Average	10.1 ± 1.0	9.9 ± 1.0	11.6 ± 2.8	11.5 ± 2.5	0.096	0.061
	ED	8.7 ± 1.1	8.6 ± 1.1	10.7 ± 3.2	10.6 ± 2.9	**0.014**	**0.016**
	ES	10.8 ± 1.2	10.8 ± 1.2	12.4 ± 2.6	12.3 ± 2.4	0.124	0.114
Perimeter, mm	Average	116.6 ± 6.5	115.9 ± 6.1	125.8 ± 13.5	125.2 ± 12.1	**0.031**	**0.038**
	ED	109.5 ± 7.4	109.4 ± 7.5	120.7 ± 15.3	120.7 ± 14.0	**0.011**	**0.009**
	ES	120.4 ± 6.7	120.0 ± 7.0	129.6 ± 12.3	128.9 ± 11.3	0.051	**0.046**
AP Diameter, mm	Average	29.5 ± 2.2	29.6 ± 2.3	30.4 ± 4.9	30.2 ± 5.3	0.454	0.280
	ED	25.0 ± 2.5	24.9 ± 2.7	28.6 ± 5.9	28.6 ± 6.2	0.056	**0.042**
	ES	32.1 ± 2.5	32.1 ± 2.5	32.5 ± 4.1	32.1 ± 4.6	0.739	0.589
IC Diameter, mm	Average	35.6 ± 2.6	35.5 ± 3.0	34.6 ± 4.7	34.6 ± 4.4	0.299	0.383
	ED	34.6 ± 2.9	34.3 ± 3.2	33.9 ± 5.3	33.6 ± 5.2	0.383	0.506
	ES	35.5 ± 2.7	35.5 ± 3.2	35.5 ± 4.3	35.6 ± 4.1	0.803	0.967
AP/IC Ratio	Average	0.83 ± 0.07	0.84 ± 0.08	0.88 ± 0.13	0.88 ± 0.14	0.135	0.244
	ED	0.73 ± 0.10	0.73 ± 0.10	0.85 ± 0.15	0.86 ± 0.17	**0.020**	**0.031**
	ES	0.91 ± 0.08	0.91 ± 0.09	0.92 ± 0.11	0.91 ± 0.13	0.589	0.868
Height, mm	Average	7.5 ± 1.6	7.7 ± 1.6	8.0 ± 1.8	8.1 ± 1.7	0.589	0.480
	ED	7.1 ± 1.6	7.3 ± 1.8	7.7 ± 1.7	7.9 ± 1.9	0.299	0.244
	ES	7.8 ± 1.5	8.3 ± 1.6	8.2 ± 1.7	8.4 ± 2.1	0.739	0.868

Data were presented as mean ± SD. *Morphological results based on 6 radially rotational slices; *P* values obtained using Mann-Whitney U test. AP: antero-posterior; IC: inter-commissural; ED: end diastole; ES: end systole.

### Reproducibility

All CMR feature tracking-derived parameters had excellent intra- and inter-observer reproducibility (Table 4), with high ICC values (all >0.9) and tight limits of agreement.

**Table 4:** Intra- and inter-observer reproducibility.

	Intra-observer			Inter-observer		
	*r*	Bias (limits of agreement)	ICC	*r*	Bias (limits of agreement)	ICC
Sm	0.981	0.04 (−0.86, 0.95)	0.990	0.967	0.06 (−1.13, 1.25)	0.982
Em	0.981	−0.01 (−1.24, 1.22)	0.991	0.957	−0.10 (−1.97, 1.78)	0.978
Am	0.984	0.05 (−0.69, 0.78)	0.992	0.974	0.10 (−0.83, 1.02)	0.987
MAPSE	0.987	0.05 (−0.95, 1.06)	0.993	0.976	0.17 (−1.20, 1.55)	0.987
3D Area	0.997	0.10 (−0.06, 0.26)	0.994	0.965	0.08 (−0.47, 0.64)	0.976
Perimeter	0.995	0.60 (−0.42, 1.63)	0.993	0.942	0.47 (−3.77, 4.71)	0.958
AP Diameter	0.985	0.25 (−0.82, 1.33)	0.984	0.979	−0.16 (−1.20, 0.89)	0.988
IC Diameter	0.949	−0.12 (−0.92, 0.67)	0.962	0.961	0.12 (−0.44, 0.68)	0.977
Height	0.989	0.01 (−0.50, 0.53)	0.993	0.980	0.06 (−0.58, 0.71)	0.989

Results were reported in Pearson’s correlation coefficient, Bland Altman analysis and intra-class correlation coefficient (ICC). (n = 10, 5 controls and 5 patients). Sm: peak systolic velocity; Em: peak early diastolic velocity; Am: peak late diastolic velocity; MAPSE: mitral annular plane systolic excursion; AP: antero-posterior; IC: inter-commissural; CI: confidence interval.

## Discussion

This is the first work to demonstrate the feasibility, reproducibility, and systematic investigation of using a feature tracking system for imaging and modeling 4D MA deformation in combination of a series of radially rotational long-axis cine CMR data with varying numbers of slices. MA dynamics and morphology were measured and extensively studied in both normal controls and patients with mitral regurgitation.

In Figs 3 and 6, Normal subjects are indicated by open circles and Patients by squares. In this way we distinguish members of the two groups while allowing a visual assessment of similarity. We used the two-sample t-test to statistically compare mean differences between CMR imaging approaches for the Normal and Patient groups as reflected in the Bland-Altman plots. Again, Normal subjects and Patients are distinguished by open circles and squares, respectively. Pearson correlation coefficients between imaging approaches were computed for Normal and Patient groups and compared using the Fisher z-statistic appropriate for comparing correlation coefficients from independent samples (see Supplementary Tables S3 and S4). In both analyses, statistical non-significance was taken as justification for combining the Normal and Patient groups into a single population to investigate the impact of different numbers of imaging slices.

MA motion serves as an important clinical indicator of regional as well as global LV function^7^. Previous attempts to assess the MA dynamics and geometry include using 2D or 3D echocardiography and computed tomography (CT)^8–13^. These approaches suffer from either limited spatial resolution and coverage^8,14,15^ or ionizing radiation^9^. With CMR evolving into a versatile, non-invasive imaging tool with excellent contrast resolution for tissue characterization, its use in automatically tracking MA motion in consecutive time frames and quantifying both dynamic and morphological changes has recently become a core component in advanced cardiac function analysis^6,16,17^. However, most of the prior studies are based on three routinely obtained long-axis cine data for MA motion tracking, and only six points for reconstructing the 3D MA^1,6^. Whether this is sufficient to constitute the ground truth has not been fully investigated. In addition, the obligatory minimal number of points for satisfactory depiction of MA dynamics and morphology had hitherto not been ascertained.

Our method involves a feature tracking algorithm with normalized cross correlation (NCC) as the similarity measure^18^. In the current application, the features within the template containing the insertion area of the mitral valve leaflets were rendered with sufficient precision to support feature matching. The majority of templates in the CMR images did not undergo highly complex deformation between adjacent time frames, granting NCC as an appropriate approach with high computational efficiency to match the template within a search region. The developed program provides a semi-automatic approach for fast localization of the MA points in each cardiac CMR slice throughout the cycle with mask selection in the initial frame as the only user input. Thereafter, the algorithm automatically executes the adaptive feature tracking for all the subsequent frames. In less than 0.3% of the total frames (120 out of 43200) in this study the target feature was not correctly tracked automatically due to temporal blurring, which necessitated manual correction. The positions of the tracked points were visually judged by expert cardiologists.

The impacts of different mask sizes and positions were tested in a small cohort. No significant differences were found regarding the mask sizes (Supplementary Table S5) and good agreements were observed for varying mask positions (Supplementary Appendix S1), indicating robustness of the tracking method, which was also demonstrated by the high reproducibility between different users and sessions for quantifications of both MA dynamics and morphology (Table 4). Second, semi-automatic CMR feature tracking without manual editing was compared with manual tracking by the experts and tissue Doppler imaging of echocardiography. Good agreements were observed as reflected by high correlation and narrow limits of agreement (Supplementary Table S6 and Fig. S1). Semi-automatic method had the advantage in time efficiency over manual tracking. The latter required approximately 10 minutes per MA point. Moreover, analysis was performed in a small cohort with multiple 9- and 6-slice sub-datasets, depending on the initial slice to be selected within the 18 slices. There were no significant differences found in terms of MA dynamics and morphology among different sub-datasets (details see Supplementary Tables S7 and S8).

The velocities and displacements derived from the MA are primarily associated with the cardiac pump function in the longitudinal direction. Motion measurements averaged from multiple MA points reflect the global function^19^. Sm and MAPSE have been shown to be good metrics of global systolic function^20^ and can detect abnormalities in patients with heart failure with preserved EF^21^. Em appears to be indicative of diastolic function and correlates well with the time constant of isovolumic relaxation^20^. A low Em/Am ratio is a good indicator of pseudonormal dysfunction even though the transmitral flow is similar to that of a normal subject^22^. Our results have shown that the mean MA motion parameters derived from feature tracking CMR are not affected by the number of uniformly selected MA points along the annulus. In particular, the MA dynamic parameters obtained from routinely acquired long-axis views are good representations and are sufficiently reliable for ventricular function assessment in clinical practice.

A conventional set of MA geometric descriptors was employed to quantify and evaluate MA morphology, viz., annular area and perimeter in 3D, minor and major diameters, and height. Prior studies have presented a broad range of normal MA dimensions. 3D echocardiographic studies have reported normal mid-systolic values ranging from 8.2 to 10.5 cm^2^ for 3D area, 106 to 128 mm for perimeter, 28 to 32.7 mm for AP diameter, 38.7 to 39 mm for IC diameter, and 6.6 to 7.9 mm for height^11–13^. Similar results have been conducted on CT, with one study reporting a mean MA area of 8.9 ± 1.5 cm^2^, perimeter of 110 ± 9 mm, AP diameter of 27.5 ± 2.7 mm and IC diameter of 37.6 ± 3.7 mm^10^. Our feature tracking CMR-derived values from the normal controls were well within these previously reported normal ranges. More importantly, the present study has for the first time investigated MA modeling with varying numbers of CMR imaging slices and demonstrated that reliable measurements of 3D MA geometry can be obtained using 6 radial slices including the routine 2-, 3- and 4-chamber views, evenly separated by 30°, centered about the mitral valve orifice and passing through the apex. These findings provide the experimental support for the use of 12 points bisecting the annulus to create a 3D model of the annulus^23,24^. In addition, the measurements of 3D area, perimeter, height, and AP diameter showed an increasing trend during systolic phase until reaching their maximal values near end of systole, which is in agreement with the findings from previous studies^12,23^.

We have compared the MA dynamics and morphology between normal controls and patients with varying degrees of mitral regurgitation. MA motion was significantly reduced in patients, which was consistent with prior results, where decreases in systolic and diastolic MA velocities were observed in patients with primary or secondary mitral regurgitation^25–27^. MA dimensions were generally enlarged in the patient group in terms of 3D area, perimeter and AP diameter. IC diameter, however, was reduced in the patient group resulting in a more circular annulus as quantified by the AP to IC diameter ratio. These results were in line with earlier studies showing patients with mitral regurgitation had increased MA size and altered MA geometry compared with normal individuals^10,17^.

In conclusion, feature tracking in long-axis cine CMR can be used to quantitatively assess 4D MA dynamics and morphology, based on either 3 routine views or 6 radially rotational slices bisecting the annulus, respectively. The yielded comprehensive and distinctive modeling characteristics may help to further study different cardiac diseases and guide surgical planning including *in vivo* evaluation of the MA prosthetic ring.

The current study has limitations. First, due to the prescribed 18 radially rotational long-axis imaging slices with a fixed 10° angular equidistance during data acquisition, available sub-datasets were constrained to only those with interslice angular distance a multiple of 10° (i.e., 9-, 6- and 3-slices datasets). For instance, a 4-slice variation with 8 equally distributed points would not have been possible, which had been shown to be effective enough for characterization of the MA dynamics and geometry in an animal study^28^.

Second, the fact that the conventional 2-, 3-, and 4-chamber views from clinical scans are prescribed not based on a simple angulation but rather on specific landmarks may be different from the setup in the present study. They may share a common point at the apex, but anatomical variations imply that they may not necessarily be co-axial or share another common point. Therefore, an additional analysis has been performed to investigate the MA dynamic measurements using the clinically conventional 2-, 3-, and 4-chamber views. As can be seen in Supplementary Table S9, good agreements were found for the clinically conventional in comparison to the radially rotational 2-, 3-, and 4-chamber views, and the 18 reference views. Nonetheless, further study is needed to investigate the impact of the prescribed conventional long-axis views in the presence of structural abnormalities (e.g. pre-existing myocardial infarction).

Third, the number of cardiac phases above 30 is typically used in 2D CMR cine for functional assessment. A lower number may not be sufficient to resolve cardiac motions, particularly at rapid systole. It is, therefore, reasonable to employ CMR feature tracking based on the standard acquisition technique, such as 40 phases per cardiac cycle that was used in the present study. An additional analysis performed based on 20 cardiac phases (downsampled from 40 phases) showed lower MA velocity and displacement (negative bias) than those based on 40 phases (Supplementary Table S10). This implies that CMR feature tracking in a dataset with a poor temporal resolution may miss an image frame that represents the point of peak velocity and therefore could systematically underestimate peak velocities.

Fourth, LV dilatation, LV hypertrophy and mitral regurgitation are often not mutually exclusive and can exist in multiple combinations. Multiple factors will cause mitral regurgitation in patients with LV hypertrophy including distorted papillary muscle, asymmetric septal hypertrophy, exaggerated systolic anterior motion, and intrinsic abnormalities of mitral valve, etc. On the other hand, the presence of mitral regurgitation increases the end diastolic volume and the ventricle has to work extra hard to pump out the increased volume at each beat. Therefore, the increased work load on ventricular muscle will lead to its hypertrophy. Both mitral regurgitation and LV hypertrophy would have impact on MA dynamics and morphology. Hence the difference in LV mass index between controls and patients may influence the comparison between the two groups. Further studies are warranted to investigate how LV hypertrophy and mitral regurgitation individually affect the MA structure and function by a longitudinal study in a large cohort where LV hypertrophy progressively develops in patients with mitral regurgitation.

There were also limitations for the proposed technique. First, the series of radially rotational long-axis cine data used for MA reconstruction were acquired sequentially. The longer than routine acquisition time may potentially introduce differences in respiratory excursion from beginning to end of acquisition. Technical advances such as 3D single-breathhold whole-heart cine imaging may overcome this problem. Second, the accuracy of NCC feature tracking can be affected by image scaling, rotation, and distortion^29^. As with other feature tracking approaches, it is dependent on there being suitable distinctive image structure around the point being tracked. Any image artifacts or enhanced noises may possibly jeopardize feature tracking results. One prior study used template matching by combining NCC and principle component analysis^30^, which could potentially improve the performance. Third, spline interpolators were used in some prior studies^6,17,23^. We used similar method to reconstruct the 3D MA ring in this study. Further studies are warranted to investigate the reliability and accuracy of a spline interpolator and how a different interpolator possibly affects the results.

The present study focused primarily on assessment of MA dynamics and morphology. A similarly comprehensive approach for the tricuspid annulus (TA) represents a potentially researchable and laudable goal in the future for evaluation of right ventricular (RV) function, as assessment by conventional 2D echocardiography remains limited due to its asymmetric, pyramidal shape^31^.

## Methods

### Study Population

Normal controls without known cardiovascular disease or other major co-morbidities, and patients with known mitral regurgitation were prospectively enrolled from two centres. The severity of mitral regurgitation was evaluated following 2003 AHA guideline using Doppler echocardiography. Patients with vena contracta width ≥0.7 cm, large central mitral regurgitation jet (area >40% left atrial area), and enlarged left ventricle were diagnosed as severe mitral regurgitation. Those with vena contracta width <0.3 cm, small central jet (area <20% left atrial area), normal left ventricular size were interpreted as mild mitral regurgitation. Meanwhile, patients with mitral regurgitation jet area in between 20–40% of left atrial area were categorized as having moderate mitral regurgitation. All subjects underwent a CMR scan. The protocol was approved by the SingHealth Centralised Institutional Review Board and the institutional review board of Shanghai Renji Hospital. Informed consent was obtained from all participants. All experiments and methods for this study were performed in accordance with relevant guidelines and regulations.

### CMR Acquisition

All subjects were imaged on a 3.0 T scanner (Ingenia, Philips Healthcare, Best, Netherlands) with a maximal 28-channel body (12-channel spine, 16-channel torso) coil, using a standard CMR protocol including multi-slice multi-direction survey images, followed by balanced fast field echo (bFFE) cine sequences in 5 short-axis slices parallel to the mitral valve ring covering the left ventricle from base to apex. Based on those, a series of 18 radially rotational slices were acquired at 10° angular equidistance in the LV long axis, extending from the apex to the centre of the mitral valve orifice (Fig. 1). For all sequences, SmartSelect functionality was used to automatically determine the coil element combination for efficient signal-to-noise ratio in the selected field-of-view (FOV) based on the coil sensitivity measured in the reference scan at the beginning of the examination for each subject^32^. Typical imaging parameters were as follows: FOV 300 × 300 mm^2^, slice thickness 8 mm, matrix size 200 × 147, acquired pixel size 1.5 × 2.0 × 8.0 mm^3^, interpolated to 1.04 × 1.04 × 8.00 mm^3^, repetition time TR 3.0 ms, echo time TE 1.5 ms, water-fat shift 0.3 pixel, 40 cardiac phases, retrospective ECG gating, partial Fourier factor 0.875, SENSE factor 2, scan time for breathhold approximately 7 seconds.

### CMR Feature Tracking

**Figure 7:**
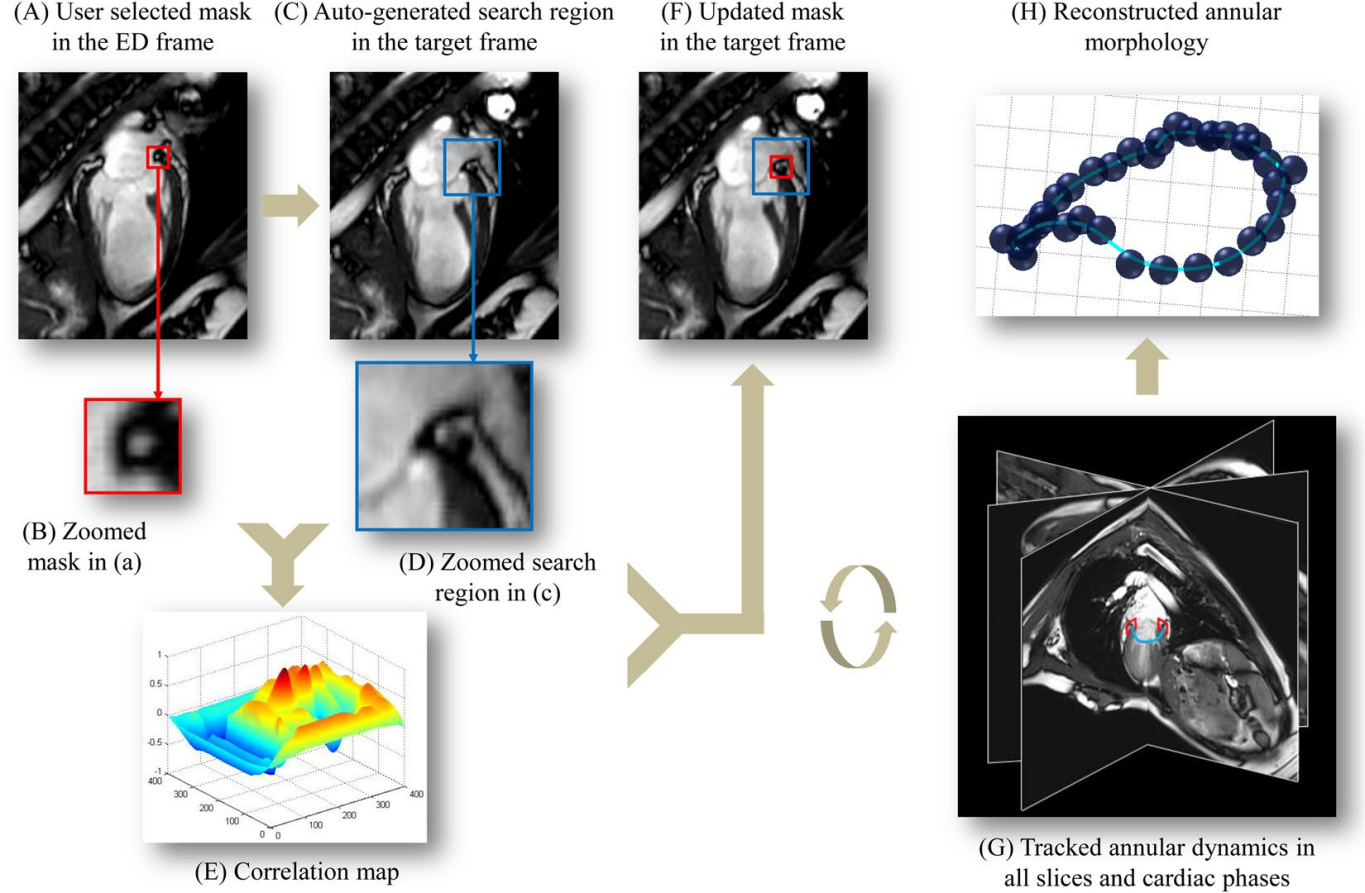
Semi-automated feature tracking algorithm on cine CMR data. (**A**,**B)** User selected mitral annulus (MA) point in the end diastolic phase of one long-axis cine-CMR series as mask (red square); **(C**,**D)** Program auto-selected search region in the target frame of the same series (blue square); **(E)** Tracking produced correlation coefficient map with the location of highest correlation coefficient corresponding to the best match; **(F)** Updated mask with located MA point in the target frame; **(G)** Tracking process iteratively executed for all frames and cine-CMR series; **(H)** Reconstructed MA ring with spatial transformation and interpolation. Details see text.

The tracking system used the method of template matching, which is an algorithm for searching and finding the location of a template image (called a mask) within a larger image (called the search region). Figure 7 outlines the procedure of applying adaptive template matching in mitral annular tracking with CMR imaging. The user manually selected the mask with size $w\times h$ in end-diastole frame of a CMR imaging sequence (usually frame 1 as shown by read rectangle in Fig. 7(A,B)). Typical size of the mask is 8 × 8 pixels. A search region sharing the same centre with the selected mask was automatically generated in frame 2 with size $(w+l)\times (h+l)$ (blue rectangle in Fig. 7(C,D)). The value of *l* was set to be 12 pixels in this study. The template matching was conducted to detect the best match of the mask in the search region by sliding the template image over the search image one pixel at a time (left to right, up to down) while computing the normalized cross correlation (NCC) at each location. Given a *w* × *h* mask **t** and the search region **x**, the matching by correlation is conducted by computing the NCC at the location $(u,v)$:

$$NCC(u,v)=\frac{\sum _{j=-h/2}^{h/2}\sum _{i=-w/2}^{w/2}{\bf{X}}(i,j){\bf{T}}(i,j)}{\sqrt{\sum _{j=-h/2}^{h/2}\sum _{i=-w/2}^{w/2}{\bf{X}}{(i,j)}^{2}}\sqrt{\sum _{j=-h/2}^{h/2}\sum _{i=-w/2}^{w/2}{\bf{T}}{(i,j)}^{2}}}$$

where

$${\bf{X}}(i,j)={\bf{x}}(u+i,v+j)-\bar{{\bf{x}}},\quad {\bf{T}}(i,j)={\bf{t}}(u+i,v+j)-\bar{{\bf{t}}}.$$

and $\bar{{\bf{x}}}$ and $\bar{{\bf{t}}}$ denote the mean value of **x** and **t**, respectively. The point with the highest correlation coefficient in the resulting correlation image (Fig. 7(E)) indicates the location of the best match. This point was used to update the mask in frame 2 that underwent the same template matching within the automatically extracted search region in frame 3 (Fig. 7(F)). This same procedure was automatically executed iteratively for all subsequent frames.

The tracking method in this study was enhanced with some latest development of an integrated software tool^33^, which is ready for clinical usage. In this software tool, we used the Insight Segmentation and Registration Toolkit (ITK) for image processing part of the MA tracking, the Visualization Toolkit (VTK) for interaction and visualization of the processed images, and Qt for graphical user interface.

### CMR Data Analysis

#### Overall framework

**Figure 8:**
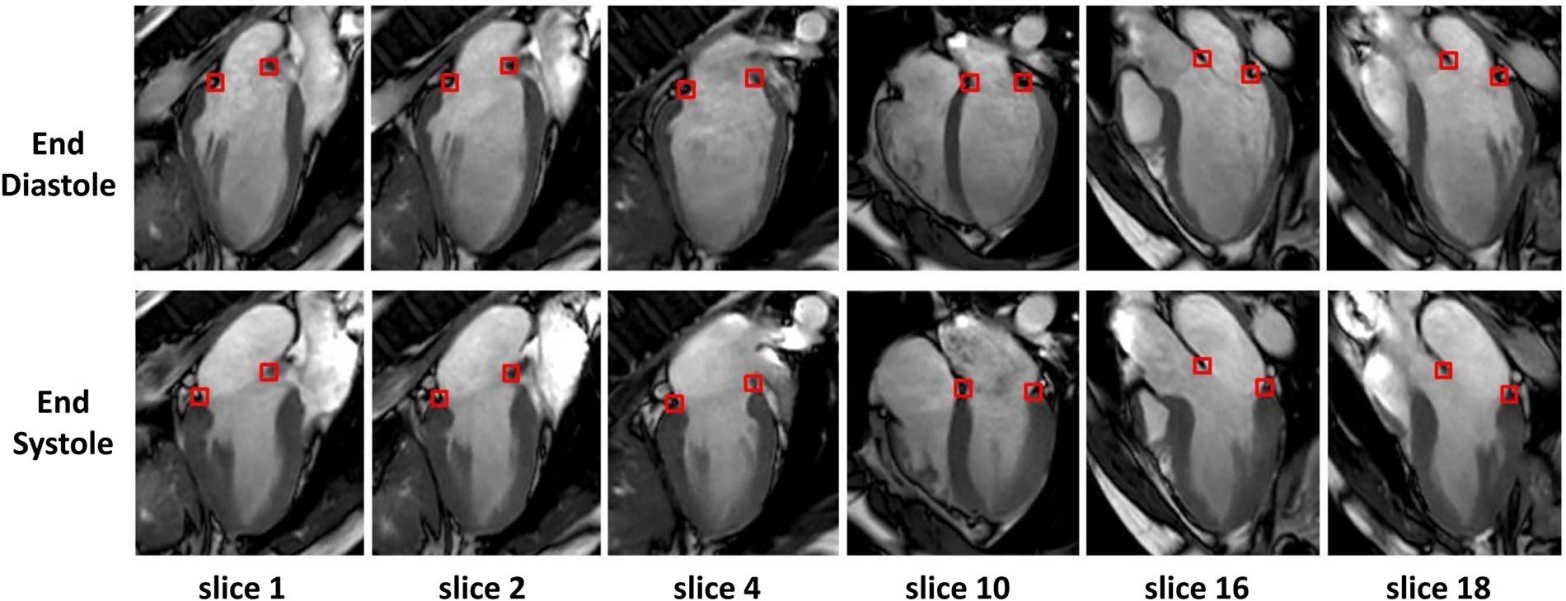
Semi-automatic feature tracking of the mitral annulus. Six series out of 18 radially rotational slices are displayed here for both end diastole (top) and end systole (bottom). Slice 4, 10 and 16 corresponded to routine 2-, 4-, and 3-chamber views, respectively, in this case.

The MA located at the junction of the left atrium and ventricle was tracked for all 18 radially rotational long-axis slices and subsequently reconstructed in 3D over the entire cardiac cycle. This was performed semi-automatically using an in-house developed program at National Heart Centre Singapore^33–35^. The whole process is shown in Fig. 7. CMR-derived MA dynamic and morphological parameters from all 36 tracking points in 18 radial slices constituted as reference. In addition, results from sparse sub-datasets with 3, 6, and 9 equally spaced slices were generated for comparison with the reference results derived from the dense full 18-slice dataset. All datasets, including the reference, contain the routinely acquired 2-, 3- and 4-chamber long-axis views (Fig. 8).

#### Motion estimation and MA dynamics

To characterize MA dynamics, two annular points in each of the 18 acquired radial slices were tracked as a function of cardiac time. As described above, MA tracking was semi-automatic with mask selection in the initial frame of each radial slices as the only user input. Figure 8 shows an example of MA tracking in selected slices.

Following the MA tracking in all 18 radial slices (Fig. 7G), four clinically useful parameters were measured on the velocity and displacement curves of each individual MA point with the averaged value to describe the MA dynamics:

- *Sm*: peak systolic velocity
- *Em*: peak early diastolic velocity
- *Am*: peak late diastolic velocity during atrial contraction
- *MAPSE*: mitral annular plane systolic excursion

#### Geometry reconstruction and MA morphology

The 2D coordinates of MA motion trajectories obtained in each of the 18 CMR slices were transformed into a 3D coordinate system. A spline curve interpolation was performed to reconstruct the 3D MA geometry, using a fully automated smoothing procedure based on a penalized least squares method^36^. The following spatiotemporal morphological measurements were calculated based on MA reconstruction for all successive frames throughout the cardiac cycle (Fig. 7H):

- *3D area*: area within the MA by triangulation with the centroid of the annulus
- *Annular perimeter*: sum up of distance between all individual contiguous points along the reconstructed annulus
- *Height*: height of the minimal annulus bounding box
- *Antero-posterior (AP) diameter*: distance between mid-points of the anterior and posterior walls.
- *Inter-commissural (IC) diameter*: distance between mid-points of the anterolateral and posteromedial walls.
- *Ratio* of AP to IC diameters.

### Reproducibility

To investigate inter-observer reproducibility, two observers performed independent analyses in a total of 10 cases (5 normal controls, 5 patients) for all MA parameters. In addition, one observer repeated the measurements of the same data in one week to assess the intra-observer reproducibility.

### Statistical Analysis

Data were summarized in mean ± SD. Comparisons of demographics and quantitative measures between the normal and patient groups were done using the Mann-Whitney U test. Agreement was assessed using Pearson’s *r* correlation, the Bland Altman graphical approach and intra-class correlation coefficient (ICC). All data analyses were performed using SAS (version 9.3, Cary, NC, USA) and SPSS (version 17.0, Chicago, IL, USA). A *P*-value of <0.05 was considered statistically significant.

We have not adjusted *P*-values for multiple testing. The family-wise error rate is the probability that one or more null hypothesis among a ‘family’ of *k* hypotheses is falsely rejected. Rather than reporting adjusted *P*-values, we propose that the reader adjust the significance level at which a given hypothesis test in the ‘family’ of hypothesis tests is rejected. Multiple approaches exist with the Bonferroni and the Bonferroni-Holm being the most familiar. Both are applicable to independent and dependent tests and are straightforward in their application when control of a ‘family-wise’ error rate is desired. The Bonferroni adjusted significance level is simply obtained by dividing the nominal alpha by the number of tests in the ‘family’. The Bonferroni-Holm is a step-wise procedure with greater power for rejecting false null hypotheses. Given these straightforward approaches for adjusting significance levels for multiple comparisons (as well as other more sophisticated methods), and in large measure owing to the certainty of disagreement among readers as to what constitutes a ‘family’, we have opted to report uncorrected *P*-values.

### Data Availability

All data generated or analyzed during this study are included in this published article (and its Supplementary Information files).

## Electronic supplementary material

Supplementary information

Supplementary Video S1 - MA tracking in all slices for a normal control

Supplementary Video S2 - MA tracking in all slices for a patient with regurgitation

Supplementary Video S3 - reconstructed MA motion with varying numbers of radial slices
