# Supplementary material for: Imaging 4D morphology and dynamics of mitral annulus in humans using cardiac cine MR feature tracking
Source: Sci Rep. 2018 Jan 8;8:81. doi: 10.1038/s41598-017-18354-2 (PMC5758818; doi:10.1038/s41598-017-18354-2)
Supplement: Supplementary file 1 — Supplementary information [file 41598_2017_18354_MOESM1_ESM.pdf]

# Imaging 4D morphology and dynamics of mitral annulus in humans using cardiac cine MR feature tracking

Shuang Leng[1†], Shuo Zhang[1,2†], Meng Jiang[3], Xiaodan Zhao[1], Rui Wu[3], John Allen[4], Ben He[3], Ru San Tan[1,4], Liang Zhong[1,4*]

[1]National Heart Research Institute Singapore, National Heart Centre Singapore, 5 Hospital Drive, Singapore 169609

[2]Philips Healthcare, Singapore 319763

[3]Department of Cardiology, Renji Hospital, School of Medicine, Shanghai Jiaotong University, Shanghai 200001, People's Republic of China

[4]Duke-NUS Medical School, 8 College Road, Singapore 169857

[†]Contributed equally

*Correspondence: Liang Zhong, zhong.liang@nhcs.com.sg

**Supplementary Table S1**. **Dynamics of mitral annulus with quantitative parameters obtained by feature tracking (n = 30, 16 controls and 14 patients).**

| Dynamic Parameters | | 18 Slices (reference) | 9 Slices (every 2) | 6 Slice (every 3) | 3 Slices* (2-, 3-, 4-C) |
|---|---|---|---|---|---|
| **Sm** | Mean (cm/s) | 6.9 | 6.9 | 7.0 | 6.8 |
| | SD (cm/s) | 1.7 | 1.7 | 1.8 | 1.7 |
| | Correlation coefficient ($r$) | - | 0.992 | 0.986 | 0.987 |
| | Mean difference (cm/s) | - | -0.021 | 0.054 | -0.114 |
| | SD of difference, $s_d$ (cm/s) | - | 0.21 | 0.30 | 0.28 |
| | Limits of agreement (cm/s) | - | (-0.44, 0.40) | (-0.54, 0.65) | (-0.66, 0.43) |
| | ICC ($r_1$) | - | 0.996 | 0.993 | 0.993 |
| | Regression equation | - | y = 1.01x-0.03 | y = 0.96x+0.22 | y = 1.00x+0.08 |
| **Em** | Mean (cm/s) | 7.4 | 7.4 | 7.5 | 7.5 |
| | SD (cm/s) | 3.5 | 3.5 | 3.6 | 3.5 |
| | Correlation coefficient ($r$) | - | 0.999 | 0.998 | 0.994 |
| | Mean difference (cm/s) | - | -0.017 | 0.011 | 0.050 |
| | SD of difference, $s_d$ (cm/s) | - | 0.17 | 0.20 | 0.40 |
| | Limits of agreement (cm/s) | - | (-0.36, 0.32) | (-0.39, 0.41) | (-0.73, 0.83) |
| | ICC ($r_1$) | - | 0.999 | 0.999 | 0.997 |
| | Regression equation | - | y = 0.99x+0.08 | y = 0.98x+0.12 | y = 1.00x-0.05 |
| **Am** | Mean (cm/s) | 7.0 | 7.0 | 7.0 | 6.9 |
| | SD (cm/s) | 2.3 | 2.3 | 2.4 | 2.3 |
| | Correlation coefficient ($r$) | - | 0.997 | 0.995 | 0.991 |
| | Mean difference (cm/s) | - | -0.001 | -0.011 | -0.051 |
| | SD of difference, $s_d$ (cm/s) | - | 0.18 | 0.25 | 0.32 |
| | Limits of agreement (cm/s) | - | (-0.35, 0.35) | (-0.50, 0.47) | (-0.67, 0.57) |
| | ICC ($r_1$) | - | 0.999 | 0.997 | 0.995 |
| | Regression equation | - | y = 1.00x+0.01 | y = 0.98x+0.15 | y = 1.01x-0.04 |
| **MAPSE** | Mean (mm) | 13.1 | 13.1 | 13.1 | 13.0 |
| | SD (mm) | 2.7 | 2.6 | 2.7 | 2.6 |
| | Correlation coefficient ($r$) | - | 0.999 | 0.997 | 0.994 |
| | Mean difference (mm) | - | -0.005 | 0.030 | -0.105 |

| | | | | | |
|---|---|---|---|---|---|
| | SD of difference, $s_d$ (mm) | - | 0.13 | 0.20 | 0.30 |
| | Limits of agreement (mm) | - | (-0.27, 0.26) | (-0.37, 0.43) | (-0.69, 0.48) |
| | ICC ($r_I$) | - | 0.999 | 0.999 | 0.997 |
| | Regression equation | - | y = 1.01x-0.15 | y = 0.99x+0.06 | y = 1.01x-0.07 |

*Clinically routinely acquired 2-, 3-, and 4-chamber long-axis views. Sm: peak systolic velocity; Em: peak early diastolic velocity; Am: peak late diastolic velocity; MAPSE: mitral annular plane systolic excursion; SD: standard deviation; ICC: intra-class correlation coefficient. Regression equation, y: 18-slices-derived results, x: 9-, 6-, and 3-slices-derived results.

**Supplementary Table S2. Morphology of mitral annulus with quantitative parameters obtained by feature tracking and geometry reconstruction (n = 30, 16 controls and 14 patients).**

| Morphological Parameters | | 18 Slices (reference) | 9 Slices (every 2) | 6 Slice (every 3) | 3 Slices* (2-, 3-, 4-C) |
|---|---|---|---|---|---|
| **3D Area** | Mean ($cm^2$) | 10.8 | 10.8 | 10.7 | 10.1 |
| | SD ($cm^2$) | 2.2 | 2.3 | 2.0 | 2.2 |
| | Correlation coefficient ($r$) | - | 0.993 | 0.985 | 0.957 |
| | Mean difference ($cm^2$) | - | 0.07 | -0.11 | -0.71 |
| | SD of difference, $s_d$ ($cm^2$) | - | 0.27 | 0.41 | 0.64 |
| | Limits of agreement ($cm^2$) | - | (-0.46, 0.61) | (-0.91, 0.69) | (-1.96, 0.54) |
| | ICC ($r_I$) | - | 0.996 | 0.990 | 0.953 |
| | Regression equation | - | y = 0.95x+0.47 | y = 1.08x-0.78 | y = 0.97x+0.99 |
| **Perimeter** | Mean (mm) | 120.9 | 122.0 | 120.3 | 115.1 |
| | SD (mm) | 11.2 | 12.1 | 10.4 | 12.0 |
| | Correlation coefficient ($r$) | - | 0.982 | 0.958 | 0.919 |
| | Mean difference (mm) | - | 1.15 | -0.64 | -5.75 |
| | SD of difference, $s_d$ (mm) | - | 2.40 | 3.22 | 4.74 |
| | Limits of agreement (mm) | - | (-3.56, 5.85) | (-6.96, 5.67) | (-15.03, 3.54) |
| | ICC ($r_I$) | - | 0.987 | 0.977 | 0.900 |
| | Regression equation | - | y = 0.91x+10.2 | y = 1.03x-3.41 | y = 0.85x+22.6 |
| **AP Diameter** | Mean (mm) | 29.9 | 30.1 | 29.8 | 30.3 |
| | SD (mm) | 3.6 | 3.7 | 3.9 | 3.4 |
| | Correlation coefficient ($r$) | - | 0.985 | 0.955 | 0.966 |
| | Mean difference (mm) | - | 0.21 | -0.09 | 0.35 |
| | SD of difference, $s_d$ (mm) | - | 0.63 | 1.17 | 0.95 |
| | Limits of agreement (mm) | - | (-1.02, 1.44) | (-2.39, 2.20) | (-1.50, 2.21) |
| | ICC ($r_I$) | - | 0.992 | 0.976 | 0.980 |
| | Regression equation | - | y = 0.98x+0.50 | y = 0.89x+3.44 | y = 1.03x-1.37 |
| **IC Diameter** | Mean (mm) | 35.1 | 35.1 | 35.1 | 33.9 |
| | SD (mm) | 3.7 | 3.6 | 3.7 | 3.9 |
| | Correlation coefficient ($r$) | - | 0.985 | 0.968 | 0.871 |
| | Mean difference (mm) | - | -0.04 | -0.07 | -1.24 |

| | | | | | |
|---|---|---|---|---|---|
| | SD of difference, $s_d$ (mm) | - | 0.63 | 0.94 | 1.95 |
| | Limits of agreement (mm) | - | (-1.28, 1.20) | (-1.91, 1.77) | (-5.06, 2.59) |
| | ICC ($r_I$) | - | 0.993 | 0.984 | 0.906 |
| | Regression equation | - | y = 1.01x-0.23 | y = 0.96x+1.41 | y = 0.81x+7.58 |
| **AP/IC Ratio** | Mean | 0.86 | 0.86 | 0.86 | 0.90 |
| | SD | 0.10 | 0.10 | 0.11 | 0.10 |
| | Correlation coefficient ($r$) | - | 0.976 | 0.933 | 0.784 |
| | Mean difference | - | 0.01 | < 0.001 | 0.04 |
| | SD of difference, $s_d$ | - | 0.02 | 0.04 | 0.07 |
| | Limits of agreement | - | (-0.04, 0.05) | (-0.08, 0.08) | (-0.09, 0.18) |
| | ICC ($r_I$) | - | 0.987 | 0.964 | 0.842 |
| | Regression equation | - | y = 1.01x-0.02 | y = 0.85x+0.13 | y = 0.79x+0.15 |
| **Height** | Mean (mm) | 7.7 | 7.8 | 7.9 | 6.8 |
| | SD (mm) | 1.7 | 1.7 | 1.6 | 2.3 |
| | Correlation coefficient ($r$) | - | 0.922 | 0.814 | 0.788 |
| | Mean difference (mm) | - | 0.12 | 0.16 | -0.96 |
| | SD of difference, $s_d$ (mm) | - | 0.67 | 1.00 | 1.44 |
| | Limits of agreement (mm) | - | (-1.19, 1.43) | (-1.80, 2.11) | (-3.78, 1.87) |
| | ICC ($r_I$) | - | 0.959 | 0.898 | 0.805 |
| | Regression equation | - | y = 0.89x+0.78 | y = 0.83x+1.18 | y = 0.56x+3.93 |

*Clinically routinely acquired 2-, 3-, and 4-chamber long-axis views. AP: antero-posterior; IC: inter-commissural; SD: standard deviation; ICC: intra-class correlation coefficient. Regression equation, y: 18-slices-derived results, x: 9-, 6-, and 3-slices-derived results.

**Supplementary Table S3. Pearson correlation coefficients and mean differences for mitral annular dynamics (18 slices vs. 3 routine slices) in normal and patient groups.**

| | **Correlation coefficient r, 18 slices vs. 3 slices (2-, 3-, 4-chamber)** | | | | **Mean difference, 18 slices vs. 3 slices (2-, 3-, 4-chamber)** | | | |
|---|---|---|---|---|---|---|---|---|
| | **All (*n* = 30)** | **Controls (*n* = 16)** | **Patients (*n* = 14)** | ***P* value*** | **All (*n* = 30)** | **Controls (*n* = 16)** | **Patients (*n* = 14)** | ***P* value#** |
| **Sm** | 0.987 | 0.978 | 0.988 | 0.294 | -0.114 | -0.082 | -0.151 | 0.504 |
| **Em** | 0.994 | 0.992 | 0.989 | 0.373 | 0.050 | 0.005 | 0.102 | 0.512 |
| **Am** | 0.991 | 0.981 | 0.990 | 0.274 | -0.051 | -0.060 | -0.041 | 0.872 |
| **MAPSE** | 0.994 | 0.989 | 0.992 | 0.378 | -0.105 | -0.110 | -0.101 | 0.942 |

*Pearson correlation coefficients computed for Normal and Patient groups and compared using Fisher z-statistic test. #Mean differences computed for Normal and Patient groups and compared using two-sample t-test. Sm: peak systolic velocity; Em: peak early diastolic velocity; Am: peak late diastolic velocity; MAPSE: mitral annular plane systolic excursion.

**Supplementary Table S4. Pearson correlation coefficients and mean differences for mitral annular morphology (18 slices vs. 6 slices) in normal and patient groups.**

| | **Correlation coefficient r, 18 slices vs. 6 slices** | | | | **Mean difference, 18 slices vs. 6 slices** | | | |
|---|---|---|---|---|---|---|---|---|
| | **All ($n$ = 30)** | **Controls ($n$ = 16)** | **Patients ($n$ = 14)** | ***P* value*** | **All ($n$ = 30)** | **Controls ($n$ = 16)** | **Patients ($n$ = 14)** | ***P* value#** |
| **3D Area** | 0.985 | 0.960 | 0.988 | 0.126 | -0.11 | -0.10 | -0.11 | 0.940 |
| **Perimeter** | 0.958 | 0.938 | 0.952 | 0.378 | -0.64 | -0.65 | -0.63 | 0.991 |
| **AP Diameter** | 0.955 | 0.941 | 0.959 | 0.360 | -0.09 | 0.02 | -0.23 | 0.583 |
| **IC Diameter** | 0.968 | 0.959 | 0.977 | 0.305 | -0.07 | -0.12 | -0.03 | 0.793 |
| **AP/IC Ratio** | 0.933 | 0.926 | 0.936 | 0.391 | < 0.001 | 0.005 | -0.007 | 0.458 |
| **Height** | 0.814 | 0.797 | 0.824 | 0.391 | 0.16 | 0.20 | 0.12 | 0.827 |

*Pearson correlation coefficients computed for Normal and Patient groups and compared using Fisher z-statistic test. #Mean differences computed for Normal and Patient groups and compared using two-sample t-test. AP: antero-posterior; IC: inter-commissural.

**Supplementary Table S5. Mitral annular dynamics derived with different mask sizes (n = 4 subjects, 2 controls and 2 patients).**

| | **Mask size 8 × 8 pixels** | **Mask size 12 × 12 pixels** | **Mask size 16 × 16 pixels** | ***P* value*** |
|---|---|---|---|---|
| **Sm** | 7.8 ± 2.1 | 7.7 ± 2.3 | 7.7 ± 2.3 | 0.875 |
| **Em** | 10.7 ± 4.2 | 10.4 ± 4.0 | 10.3 ± 3.8 | 0.676 |
| **Am** | 7.9 ± 2.2 | 7.9 ± 2.3 | 8.0 ± 2.4 | 0.945 |
| **MAPSE** | 14.7 ± 2.7 | 14.8 ± 2.9 | 14.7 ± 2.7 | 0.940 |

Data were presented as mean ± SD. Sm: peak systolic velocity; Em: peak early diastolic velocity; Am: peak late diastolic velocity; MAPSE: mitral annular plane systolic excursion; **P* value obtained by one-way ANOVA test.

**Supplementary Table S6. Comparisons between semi-automatic and manual tracking results (n = 4, 2 controls and 2 patients).**

| Variables | Technique | Mean | Bias (limits of agreement) | *P* value* |
|---|---|---|---|---|
| **Sm** | Manual | 8.2 ± 2.2 | | |
| | Semi-automatic | 7.8 ± 2.1 | -0.4 (-1.5, 0.8) | 0.144 |
| **Em** | Manual | 11.0 ± 4.2 | | |
| | Semi-automatic | 10.7 ± 4.2 | -0.3 (-1.5, 1.0) | 0.609 |
| **Am** | Manual | 8.1 ± 2.3 | | |
| | Semi-automatic | 7.9 ± 2.2 | -0.2 (-1.6, 1.1) | 0.409 |
| **MAPSE** | Manual | 14.9 ± 2.8 | | |
| | Semi-automatic | 14.7 ± 2.7 | -0.2 (-1.1, 0.7) | 0.579 |
| **3D Area** | Manual | 9.12 ± 1.40 | | |
| | Semi-automatic | 9.08 ± 1.37 | -0.04 (-0.09, 0.01) | 0.814 |
| **Perimeter** | Manual | 110.48 ± 8.56 | | |
| | Semi-automatic | 110.41 ± 8.51 | -0.07 (-0.44, 0.30) | 0.943 |
| **AP Diameter** | Manual | 28.11 ± 2.91 | | |
| | Semi-automatic | 28.06 ± 2.87 | -0.05 (-0.22, 0.12) | 0.901 |
| **IC Diameter** | Manual | 33.71 ± 1.44 | | |
| | Semi-automatic | 33.65 ± 1.42 | -0.06 (-0.25, 0.13) | 0.742 |
| **Height** | Manual | 7.05 ± 1.12 | | |
| | Semi-automatic | 6.94 ± 1.05 | -0.11 (-0.26, 0.04) | 0.349 |

Sm: peak systolic velocity; Em: peak early diastolic velocity; Am: peak late diastolic velocity; MAPSE: mitral annular plane systolic excursion; AP: antero-posterior; IC: inter-commissural. **P* value obtained by two-sample t-test.

**Supplementary Table S7. Mitral annular dynamics derived using multiple sub-datasets (n = 4, 2 controls and 2 patients).**

| | Sm, cm/s | Em, cm/s | Am, cm/s | MAPSE, mm |
|---|---|---|---|---|
| **18 slices** | 7.8 ± 2.1 | 10.7 ± 4.2 | 7.9 ± 2.2 | 14.7 ± 2.7 |
| **9 slices (slice no.: 1, 3,…,17)** | 7.8 ± 2.1 | 10.6 ± 4.1 | 7.8 ± 2.2 | 14.7 ± 2.7 |
| **9 slices (slice no.: 2, 4,…,18)** | 7.8 ± 2.0 | 10.8 ± 4.4 | 8.0 ± 2.1 | 14.7 ± 2.8 |
| **6 slices (slice no.: 1, 4,…,16)** | 7.7 ± 2.1 | 10.7 ± 4.2 | 7.9 ± 2.2 | 14.8 ± 2.8 |
| **6 slices (slice no.: 2, 5,…,17)** | 7.8 ± 2.0 | 10.9 ± 4.3 | 8.0 ± 2.1 | 14.7 ± 2.7 |
| **6 slices (slice no.: 3, 6,…,18)** | 8.0 ± 2.1 | 10.5 ± 4.2 | 7.8 ± 2.2 | 14.7 ± 2.8 |
| **3 slices (2-, 3-, 4-chamber)** | 7.6 ± 2.0 | 10.7 ± 4.0 | 8.1 ± 2.1 | 14.6 ± 2.6 |
| ***P* value*** | 0.988 | 0.999 | 0.954 | 0.998 |

Data were presented as mean ± SD. Sm: peak systolic velocity; Em: peak early diastolic velocity; Am: peak late diastolic velocity; MAPSE: mitral annular plane systolic excursion. **P* value obtained by one-way ANOVA test.

**Supplementary Table S8. Mitral annular morphology derived using multiple sub-datasets (n = 4, 2 controls and 2 patients).**

| | 3D Area, $cm^2$ | Perimeter, mm | AP Diameter, mm | IC Diameter, mm | Height, mm |
|---|---|---|---|---|---|
| **18 slices** | 9.1 ± 1.4 | 110.4 ± 8.5 | 28.1 ± 2.9 | 33.7 ± 1.4 | 6.9 ± 1.0 |
| **9 slices (slice no.: 1, 3,…,17)** | 9.2 ± 1.6 | 111.7 ± 11.1 | 28.4 ± 3.1 | 33.4 ± 1.6 | 6.9 ± 1.0 |
| **9 slices (slice no.: 2, 4,…,18)** | 9.2 ± 1.3 | 110.9 ± 8.3 | 28.8 ± 2.6 | 33.5 ± 1.5 | 7.3 ± 1.2 |
| **6 slices (slice no.: 1, 4,…,16)** | 9.0 ± 1.3 | 109.3 ± 7.5 | 28.4 ± 2.8 | 33.7 ± 1.6 | 7.0 ± 0.8 |
| **6 slices (slice no.: 2, 5,…,17)** | 9.0 ± 1.3 | 108.6 ± 8.3 | 29.7 ± 2.9 | 33.1 ± 1.6 | 6.7 ± 1.3 |
| **6 slices (slice no.: 3, 6,…,18)** | 9.4 ± 1.4 | 111.3 ± 9.4 | 30.1 ± 2.5 | 33.6 ± 1.4 | 7.4 ± 1.0 |
| ***P* value*** | 0.944 | 0.959 | 0.685 | 0.951 | 0.668 |

Data were presented as mean ± SD. AP: antero-posterior; IC: inter-commissural. *$P$ value obtained by one-way ANOVA test.

**Supplementary Table S9. Mitral annular dynamics derived using conventional two-, three-, and four-chamber views.**

| | Variables | *r* | Bias (limits of agreement) | *P* value* |
|---|---|---|---|---|
| **Conventional vs. Radially rotational 2-, 3-, and 4-chamber views** | **Sm** | 0.975 | 0.05 (-0.57, 0.68) | 0.916 |
| | **Em** | 0.980 | -0.37 (-1.60, 0.86) | 0.723 |
| | **Am** | 0.982 | 0.22 (-0.53, 0.98) | 0.730 |
| | **MAPSE** | 0.959 | -0.12 (-1.22, 0.97) | 0.855 |
| **Conventional 2-, 3-, and 4-chamber views vs. 18 reference views** | **Sm** | 0.953 | -0.32 (-1.19, 0.55) | 0.537 |
| | **Em** | 0.954 | 0.32 (-1.46, 2.10) | 0.760 |
| | **Am** | 0.952 | -0.35 (-1.49, 0.79) | 0.590 |
| | **MAPSE** | 0.925 | 0.01 (-1.45, 1.47) | 0.986 |

Sm: peak systolic velocity; Em: peak early diastolic velocity; Am: peak late diastolic velocity; MAPSE: mitral annular plane systolic excursion; r: Pearson correlation coefficient; **P* value obtained by one-way ANOVA test.

**Supplementary Table S10. Mitral annular dynamics derived using all radial slices with different numbers of cardiac phases.**

| | Variables | *r* | Bias (limits of agreement) | *P* value* |
|---|---|---|---|---|
| **20 vs. 40 cardiac phases** | **Sm** | 0.980 | -0.61 (-1.48, 0.26) | 0.043 |
| | **Em** | 0.965 | -1.44 (-3.92, 1.04) | 0.011 |
| | **Am** | 0.917 | -1.48 (-3.18, 0.22) | < 0.001 |
| | **MAPSE** | 0.993 | -0.21 (-0.85, 0.44) | 0.587 |

Sm: peak systolic velocity; Em: peak early diastolic velocity; Am: peak late diastolic velocity; MAPSE: mitral annular plane systolic excursion; r: Pearson correlation coefficient; **P* value obtained by two-sample t-test.

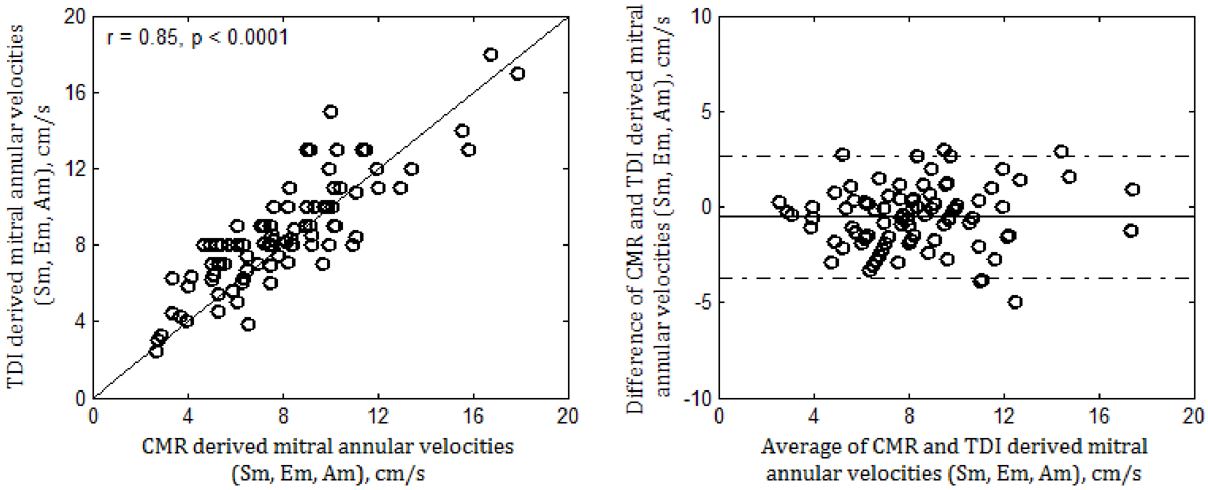


**Supplementary Figure S1. Comparison of CMR and TDI derived mitral annular velocities.**

**Supplementary Video S1. Feature tracking of 4D MA dynamics in a normal control subject in all 18 cine CMR slices, each with two annular points over the entire cardiac cycle.** Details see text.

**Supplementary Video S2. Feature tracking of 4D MA dynamics in a patient with mitral regurgitation in all 18 cine CMR slices, each with two annular points over the entire cardiac cycle.** Details see text.

**Supplementary Video S3. Feature tracking of 4D MA morphology in a normal control in cine CMR with different numbers of equidistant radially rotational long-axis slices.** These include all 18 slices, 9 slices (every other slices), 6 slices (every three slices), and 3 slices (routine 2-, 3-, 4-chamber views), respectively. Dots indicate intersection points along the mitral valve orifice, which is represented by the interpolated curve. Details see text.

**Supplementary Appendix S1 – Robustness evaluation of the tracking method**

To evaluate the robustness of the tracking method, analysis has been performed in a subset of patients and normal controls with two subjects in each category to test the correspondence of feature tracking via slight variations on the initial position around the feature of interest.

The robustness testing for each point around the mitral annulus was conducted as follows:

1) The position of original mask in the initial frame is denoted as $(x_o, y_o)$. The size of the mask is $w \times h$.
2) The new mask position $(x_n, y_n)$ was obtained by slightly varying the original as $(x_n = x_o + \Delta w, y_n = y_o + \Delta h)$, where $\Delta w$ and $\Delta h$ were randomly generated between $-0.15\Delta w$ and $+0.15\Delta w$, and between $-0.15\Delta h$ and $+0.15\Delta h$, respectively.
3) Semi-automatic tracking was executed with the new mask without manual intervention.
4) Mitral annular dynamic and morphological measurements were then derived and compared with the original results.

The comparison results are given in the table below. Good agreements were observed for all mitral annular measurements between the two sets of results, indicating good robustness of the tracking method.

**Supplementary Table S11. Robustness test results by comparing measurements from slight variation of mask position with original measurements.**

| | Variables | r | Bias (limits of agreement) | ICC |
|---|---|---|---|---|
| Original results vs. Results by slight variation of mask position | Sm | 0.990 | 0.01 (-0.58, 0.60) | 0.995 |
| | Em | 0.989 | 0.02 (-1.23, 1.26) | 0.994 |
| | Am | 0.961 | -0.10 (-1.30, 1.09) | 0.980 |
| | MAPSE | 0.984 | -0.08 (-1.05, 0.88) | 0.991 |
| | 3D Area | 0.997 | 0.13 (-0.09, 0.35) | 0.996 |
| | Perimeter | 0.980 | 1.47 (-2.30, 5.24) | 0.977 |
| | AP Diameter | 0.991 | -0.22 (-0.99, 0.55) | 0.994 |
| | IC Diameter | 0.943 | 0.63 (-0.30, 1.55) | 0.923 |
| | AP/IC Ratio | 0.947 | 0.08 (-0.81, 0.97) | 0.961 |

Sm: peak systolic velocity; Em: peak early diastolic velocity; Am: peak late diastolic velocity; MAPSE: mitral annular plane systolic excursion; AP: antero-posterior; IC: inter-commissural; r: Pearson correlation coefficient; ICC: intra-class correlation coefficient.
